# TRPV4 and PIEZO Channels Mediate the Mechanosensing of Chondrocytes to the Biomechanical Microenvironment

**DOI:** 10.3390/membranes12020237

**Published:** 2022-02-18

**Authors:** Min Zhang, Nan Meng, Xiaoxiao Wang, Weiyi Chen, Quanyou Zhang

**Affiliations:** 1 College of Biomedical Engineering, Taiyuan University of Technology, Taiyuan 030024, China; zhangmin0966@link.tyut.edu.cn (M.Z.); mengnan1137@link.tyut.edu.cn (N.M.); wangxiaoxiao1290@link.tyut.edu.cn (X.W.); 2 Shanxi Key Laboratory of Bone and Soft Tissue Injury Repair, Department of Orthopedics, The Second Hospital of Shanxi Medical University, No. 382, Wuyi Road, Taiyuan 030001, China

**Keywords:** chondrocyte, mechanical microenvironment, mechanotransduction, calcium signaling, TRPV4, PIEZOs

## Abstract

Articular cartilage and their chondrocytes are physiologically submitted to diverse types of mechanical cues. Chondrocytes produce and maintain the cartilage by sensing and responding to changing mechanical loads. TRPV4 and PIEZOs, activated by mechanical cues, are important mechanosensing molecules of chondrocytes and have pivotal roles in articular cartilage during health and disease. The objective of this review is to introduce the recent progress indicating that the mechanosensitive ion channels, TRPV4 and PIEZOs, are involved in the chondrocyte sensing of mechanical and inflammatory cues. We present a focus on the important role of TRPV4 and PIEZOs in the mechanotransduction regulating diverse chondrocyte functions in the biomechanical microenvironment. The review synthesizes the most recent advances in our understanding of how mechanical stimuli affect various cellular behaviors and functions through differentially activating TRPV4 and PIEZO ion channels in chondrocyte. Advances in understanding the complex roles of TRPV4/PIEZO-mediated mechanosignaling mechanisms have the potential to recapitulate physiological biomechanical microenvironments and design cell-instructive biomaterials for cartilage tissue engineering.

## 1. Introduction

Articular cartilage is routinely exposed to a wide array of dynamic mechanical loading, including compressive, tensile strains, fluid shear, and osmolality [1]. Chondrocytes, the sole cell type comprising articular cartilage, are exposed to a variety of mechanical stimuli during physiological and pathological joint loading [2,3], and thus the ability to perceive and respond properly to these signals is critical for the maintenance of joint health [4]. In vivo, chondrocytes reside within the structurally distinctive pericellular matrix (PCM), which is pivotal in transmitting biomechanical and biological signals between the extracellular matrix (ECM) and chondrocytes [5]. The changes of mechanical cues in PCM composition can obviously affect mechanotransduction in chondrocytes [6]. At present, it is generally agreed that the mechanosensory mechanisms by which cells grow, adapt, and respond to mechanobiological signals in their mechanical microenvironment, partly through mechanosensitive ion channels, integrins, the primary cilium, matrix protein receptors, cell adhesion molecules, and cytoskeletal elements and, more importantly, through the putative mechanically activated ion channels, such as transient receptor potential vanilloid 4 (TRPV4) and PIEZOs [7,8,9,10].

The biomechanical microenvironment of chondrocytes has an important impact on cartilage homoeostasis and function [11]. Chondrocytes use specialized mechanisms to sense and respond to a change in their biomechanical microenvironment through mechanotransduction processes, which convert the mechanical signals into a biochemical response. Chondrocytes sensing mechanical stimuli are critical to cartilage homeostasis and osteoarthritis development. The earliest responses in chondrocyte mechanotransduction pathways involve calcium influx and changes in the mitochondrial function, which occur in seconds to minutes. Chondrocyte mechanosensation involves an integrated set of pathways that may also include the transmission of pericellular mechanical and osmotic challenges to the cell and nucleus via integrins and various cytoskeletal components. A large number of researchers insist that intracellular mechanobiology was largely thought to be mediated by transmembrane proteins, such as integrins, but the emerging role of the mechanically activated ion channels in sensing external mechanical cues can potentially remodel our present understanding.

Mechanically activated ion channels have been gaining widespread attention from the research community as key mechanosensors that convert mechanical stimuli to cellular responses by exciting cell membranes or triggering calcium signaling [12]. Furthermore, mechanical sensitive channels represent a specialized type of mechanotransducers, which can rapidly respond to changes of mechanical cues. Mechanically activated ion channels have been proposed as sensors of physical force, but the molecular identity of these mechanosensitive channels and an understanding of how mechanical force is transduced have remained elusive for decades, significantly hampering our understanding of the physiological significance of mechanotransduction in vivo. Chondrocytes express an array of mechanically sensitive ion channels and receptors, including TRPV4 and PIEZO channels, integrins, and primary cilia [13]. However, among such mechanosensors, we only focus on PIEZO/TRPV4-mediated mechanotransduction by which mechanical cues are transduced by chondrocytes. Mechanosensitive activation of TRPV4 and PIEZO channels is critical in the development, remodeling, and homeostasis of articular cartilage.

Extracellular calcium influx is central to the response of cells to mechanical loading [14]. Changes in Ca^2+^ concentration are mainly realized through calcium channels on the membrane. The regulation of the intracellular free Ca^2+^ concentration is a key factor in the process of information transfer. Ca^2+^ signaling regulates many processes in chondrocytes including division, migration, death, and differentiation [15]. In addition, the cartilage traumatic injury-induced chondrocytic Ca^2+^ signal impacts the cytoskeletal structure, energy homeostasis, apoptotic equilibrium, and inflammatory phenotype [16,17,18,19,20]. The concentration of cytoplasmic Ca^2+^ can be significantly increased when cells are stimulated by physical and chemical factors [12,21]. While several ion channels are expressed in chondrocytes that could affect calcium signaling, TRPV4 and PIEZOs, the two types of mechanosensitive channels, are of great concern and have been identified in chondrocytes and preferentially conduct calcium [22]. TRPV4 and PIEZOs are two of the most important ion channels, and both functionally express to play a largely anabolic role in matrix biosynthesis by articular chondrocytes. PIEZO1 or TRPV have been shown to improve bone formation, cartilage tissue formation, and their mechanical properties by mimicking the effects of mechanical loading [9,23].

The aim of this review is to provide an overview of current knowledge of TRPV4 and PIEZOs and discuss their critical role in the mechanotransduction of diverse cell functions in physiological and pathological conditions. A deeper understanding of these events can elucidate new therapeutic targets for early intervention to prevent osteoarthritis.

## 2. PIEZOs and TRPV4 in Chondrocyte

**Figure 1:**
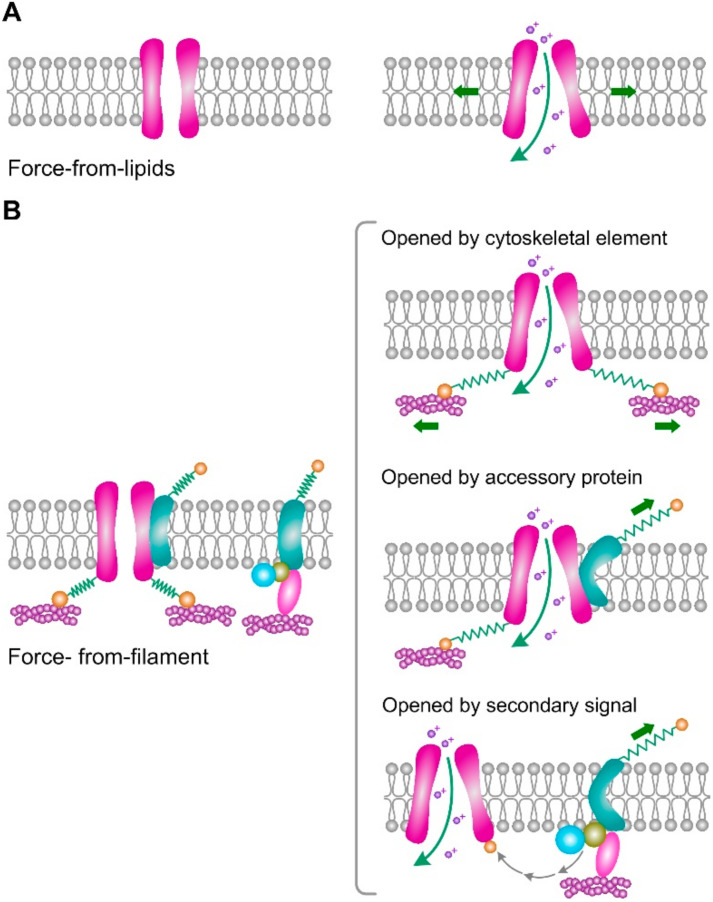
Two general models of channel gating by mechanical stimuli. (**A**) In the force-from-lipids model, surface tension of the membrane causes a hydrophobic mismatch that opens the channel. (**B**) In the force-from-filament model, specific accessory proteins are bound to the channel, mechanical stimulus, or secondary signal produced by these proteins can open channel. Figure based on reference [24].

Mechanically sensitive ion channels mediate a vast array of different cellular and organismic sensations. How they convert a force stimulus into channel gating has remained largely a mystery. PIEZO and TRPV4 channels have been implicated in a wide variety of mechanical transduction processes in articular cartilage and chondrocyte [7,10]. The activation of PIEZO and TRPV4 channels requires the coupling of the channel to a mechanical stimulus. This coupling eventually can convert diverse forms of mechanical force into local deformation, which can lead to ion channel conformational change and gating in response to membrane stretching and deformations. Two models have been suggested for the mode of activation of mechanically activated channels: force-from-lipids and force-from-filament (Figure 1) [24]. In the force-from-lipids model, the gating force is delivered to the channel by surface tension or bending of the membrane, which interacts with the surrounding lipids and opens the channel. In the force-from-filament model, the gating force is coupled via a proteinaceous tether that links the channel to specific structural components that are ECM molecules or intracellular cytoskeletal elements. In addition to the above two cases, dual tethering mechanisms have been proposed where tethers link to structural elements on both sides of the membrane.

The PIEZO family of genes, including PIEZO1 and PIEZO2, are pore-forming subunits of ion channels and identified as bona fide mechanically activated Ca^2+^-permeable cation channels with comparatively faster inactivation kinetics [25,26]. PIEZO1 shows widespread expression and confers mechanosensitivity in many different types of cells [27]. The PIEZOs can sense diverse forms of mechanical stimuli and be directly activated by mechanical force or deformation. Even mechanical perturbations of the lipid bilayer alone are sufficient to activate PIEZO channels, illustrating their innate ability as molecular force transducers [25,26,27,28,29]. Growing evidence has shown that PIEZOs play an important role in the mechanoregulation of a wide range of physiological and pathological functions. TRPV4 channels, first cloned repeatedly by following hypotonicity-induced Ca^2+^ signals, have been implicated in a wide variety of mechanosensory processes [30,31]. TRPV4 is a nonselective cation channel that is calcium permeable and shows polymodal activation by diverse mechanical stimuli including substrate elastic and viscoelastic, cell swelling, and dynamic strain [32,33]. Distinct from Piezo1, TRPV4 channels play prominent roles in regulating the intracellular Ca^2+^ concentration in nonexcitable cells. So far, most evidence suggests that TRPV4 lies downstream of an osmotic sensor and mediates the transduction of osmotic stimuli in a diverse range of cell types. However, how osmotic force activates TRPV4 remains unknown. Among the Ca^2+^-permeable transient receptor potential channels, TRPV4 has been studied extensively for its response to diverse mechanical and osmotic stimuli depending on the cell type and context [34]. To date, whether TRPV4 is directly gated or indirectly activated by mechanical stimuli remains unclear. Articular chondrocytes, which are physiologically nonneural mechanosensitive cells, functionally express TRPV4 and PIEZOs [35]. Despite the molecular structure of PIEZO1 and TRPV4, they are found in several mechanically sensitive cells [36,37], and the underlying downstream signal transduction pathways mediated by PIEZO1 and TRPV4 are not clear.

## 3. TRPV4 and PIEZOs Mediate the Chondrocyte Sensing of Mechanical and Inflammatory Cues

Cellular mechanotransduction is essential for sensing external mechanical stimuli in the mechanical microenvironment relevant to physiological and disease states. In articular cartilage, chondrocytes are constantly exposed to exogenous and endogenous forces that are referred to as biomechanical cues from the PCM. The mechanical loading of cartilage induces a wide range of physical stimuli to which chondrocytes are sensitive, including changes in extracellular and pericellular matrix strain, pH, fluid sheer stress, hydrostatic pressure, and streaming potentials [38]. Given the ever-growing importance of the PCM, it is now universally accepted that mechanotransduction is generally important for chondrocytes and regulates their mechanobiological processes as diverse as cell migration, proliferation, and differentiation. At the core of these events is the conversion of biomechanical cues into biochemical signals, which is accomplished mainly by the activation of mechanosensitive ion channels.

Previous studies have identified that TRPV4 and PIEZOs function synergistically in chondrocyte mechanotransduction in response to injurious mechanical loading [8,9]. TRPV4-mediated Ca^2+^ influx regulates SOX9 expression [39], a key transcription factor involved in cartilage matrix synthesis. TRPV4 agonist-treated constructs derived from primary bovine articular chondrocytes increase collagen content and tensile stiffness [40]. Additionally, TRPV4 activation also influences morphological and biochemical properties of neocartilage constructs [41]. A recent study used a mechanically responsive bioartificial tissue construct to redirect endogenous mechanically sensitive TRPV4 to drive synthetic genetic circuits for converting mechanical inputs into a programmed expression of a therapeutic transgene [42]. This groundbreaking work provided promising autonomous therapeutics and drug delivery systems for osteoarthritis and other age-related joint.

TRPV4 mechanosensitivity is closely related to the type of mechanical stimulation [43]. Thus, the complex mechanical cues of the cellular microenvironment may activate TRPV4 channels by distinct mechanisms and to different extents. For example, the regulation of TRPV4 activation in response to cyclic tensile strain, arachidonic acid release, and membrane deformation is obviously different [43,44,45]. Another important role of TRPV4 activation in the anti-inflammatory mechanism of mechanical stimulation indicates that mechanically or pharmaceutically activated TRPV4 inhibits proinflammatory IL-1β signaling and cartilage degradation through the regulation of HDAC6 and ciliary tubulin [44]. Further research also showed that IL-1α sensitizes chondrocytes to injurious loading through PIEZO1-mediated calcium signaling [46]. However, TRPV4 was not involved in this pathway. Thus, this inflammatory sensitization to mechanical stimulation may be selective. Several reports have suggested that actin and microtubules may be closely associated with TRPV4 channels [47,48,49]. A recent report indicated that IGF-1 regulates TRPV4-mediated Ca^2+^ flux in chondrocytes via reorganization of the actin cytoskeleton in response to osmotic stimuli [50]. Additionally, a TRPV4-mediated Ca^2+^ influx might induce chondrocyte apoptosis, while high-level mechanical stress upregulated levels of TRPV4 [51]. Thus, it is possible that attenuating TRPV4 levels or activity might produce a protective effect against cartilage degeneration.

PIEZO1 is characteristically activated by various kinds of mechanical stimulation, including poking, stretching, shear stress, and substrate deflection [25,43,52]. So far, PIEZO channels have been reported to extensively participate in various mechanotransduction processes in diverse tissues and cell types [27]. In articular cartilage, the function of the PIEZO channel, as the major mechanotransduction, is responsible for regulating calcium signaling and maintaining the cartilage matrix. The skeletal system is exposed to extensive mechanical loading. In some but not all species, PIEZO2 contributes to signaling in chondrocytes. PIEZO1 and PIEZO2 together mediate calcium signals in porcine chondrocytes when compressed at hypertrophic levels by atomic force microscopy (AFM) [9]. Murine chondrocytes express detectable levels of PIEZO1 (but not PIEZO2) and TRPV4 protein, and these channels together mediate the mechanically activated current when the cell–matrix interface is stimulated [43]. Additionally, cytoskeletal proteins determining membrane stiffness regulate PIEZO activity. For example, the changes of membrane stiffness induced by dynamin in chondrocytes can alter PIEZO1 activity [9]. Chondrocyte death acts as a critical mechanism leading to joint injury-induced post-traumatic arthritis following trauma [53,54,55]. Thus, TRPV4 and PIEZOs could potentially provide molecular targets for reducing cell death and mitigating injury-induced cartilage degeneration. However, cellular targets that initiate protective or restorative signaling pathways induced by TRPV4 and PIEZO channels need further clinical study.

**Figure 2:**
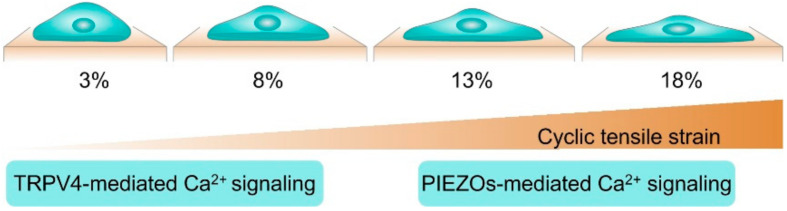
TRPV4 and PIEZO-mediated calcium oscillation in chondrocyte during cyclic tensile strain stimulation. Figure based on our recent publication [56].

Our study indicated that TRPV4-mediated Ca^2+^ signaling played a central role in the response of chondrocytes to low-strain (physiological) levels of strain (3% and 8% of strain), while PIEZO2-mediated Ca^2+^ signaling played a central role in the response of chondrocytes to high-strain (traumatic) levels (18% of strain) [56] (Figure 2). Consistent with our results, a seminar study also discovered that the inhibition of the TRPV4 channel protects against aging-associated OA but not against traumatic mechanically induced OA [23]. Collectively, these studies raise the possibility of therapeutically targeting TRPV4-mediated mechanotransduction for the matrix formation and OA treatment. Growing evidence suggests that TRPV4 plays an important role in mediating cellular and tissue inflammation, and studies of chondrocyte-specific TRPV4 knockout mice reported a decreased severity of age-associated osteoarthritis [57]. Hattori et al., for the first time, demonstrated that TRPV4 activation contributed to the chondroprotective effect by activating CaMKK/AMPK and suppressing the activation of NF-κB [58]. Despite the fact that the exact mechanism of the AMPK activation suppressing NF-κB phosphorylation remains unclear, it is well known that AMPK-associated signaling pathways inhibit IL-1β-induced inflammatory responses by inhibiting NF-κB activation in chondrocytes. Collectively, these studies may offer a promising therapeutic option for the matrix formation and OA treatment.

## 4. TRPV4 and PIEZOs Mediate the Chondrocyte Sensing of Physical Signals of the Cellular Microenvironment

**Figure 3:**
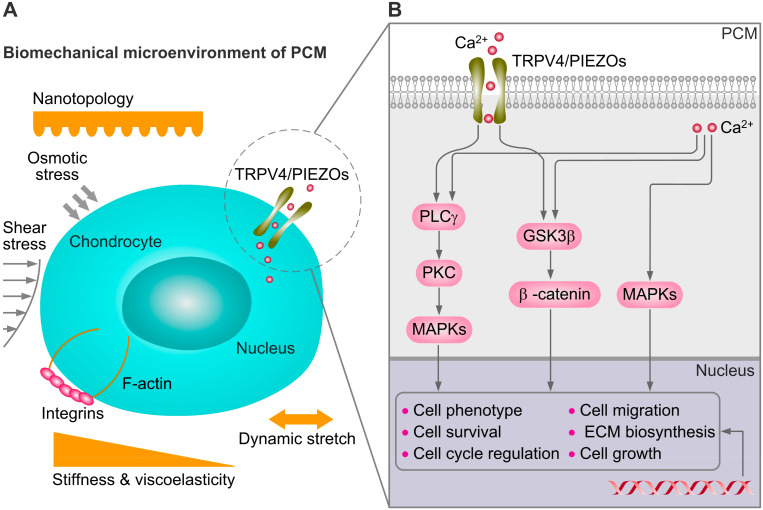
Schematic of mechanical microenvironment cues and mechanoregulating of the chondrocyte. The PCM is characterized by the presence of collagens type VI and IX. The mechanical properties of PCM, combined with several other types of mechanical cues, determine the mechanical microenvironment of chondrocytes. PCM can serve as a transducer of the biomechanical cues through regulation of dynamic stretch, compression, osmotic, and fluid shear stress of the chondrocyte. Subfigures (**A**,**B**) based on reference [6,60].

The mechanical microenvironment of chondrocytes is prominently represented in numerous physiological as well as pathophysiological processes in the different stages of development and disease. Chondrocytes can sense variations in substrate stiffness through TRPV4 and PIEZO1 and downstream activation of transcription factor activity [5,59]. Thus, the recapitulation of the mechanical properties (substrate stiffness, viscoelastic, plasticity, topography, and microniche geometry) of the native PCM is necessary to understand the mechanoregulating mechanism of TRPV4 and PIEZOs in chondrocyte (Figure 3).

During the last decades, bioengineers have developed a number of suitable state-of-the-art techniques, such as micropatterning, electrospinning, and organ-on-a-chip technology [61]. These approaches can be used to recapitulate the PCM microniche with its biomechanical and biological cues in vitro. Various mechanical cues of the cellular microenvironment have been engineered in vitro to investigate their roles in regulating cellular behaviors both spatially and temporally. Among all these mechanical cues of the cellular microenvironment, osmotic pressure [62], substrate stiffness/viscoelasticity [59], and mechanical forces are the three main aspects of the mechanical environment for regulating cell mechanobiological behavior (Figure 3). Extracellular matrix (ECM) stiffness, viscoelasticity, and microniche geometry, as important biomechanical cues of the cellular microenvironment, are widely reviewed [59,60,63]. Of interest, emerging evidence supports that TRPV4 and PIEZOs are involved in the mechanoregulation during the chondrocyte sensing of mechanical microenviromental cues. TRPV4 and PIEZO channels initiate calcium signaling, which is involved in chondrocyte mechanotransduction and interacts with multiple downstream pathways including phospholipase Cγ (PLCγ), glycogen synthase kinase-3β (GSK3β), and mitogen-activated protein kinases (MAPKs) that are relevant for OA (Figure 3) [6].

A recent study showed that chondrocytes can promoted a striking increase in volumes of the interconnected cartilage matrix in 3D viscoelastic hydrogels with fast stress relaxation [10]. A previous study indicated that reciprocal feedback between TRPV4 activation and volume expansion mediated the osteogenic commitment of mesenchymal stem cells in 3D viscoelastic matrices [64]. Notably, this sensing of matrix stiffness by the chondrocytes was dependent on calcium signaling. Inhibiting TRPV4-mediated calcium signaling led to the inability of healthy chondrocytes to respond to matrix stiffness [65]. In OA chondrocytes, the notably deficient in sensing matrix mechanical properties led to incompetent mechanically driven homeostasis and an inability of tissue remodeling [66]. The above-mentioned work suggested that TRPV4 had an important role in cartilage extracellular matrix homeostasis. However, our mechanistic understanding of mechanotransduction in viscoelastic matrices in three dimensions is still limited. It is not clear yet how TRPV4 and PIEZO channels are involved in matrix viscoelasticity affecting signaling pathways and the regulation of transcription in three dimensions.

**Figure 4:**
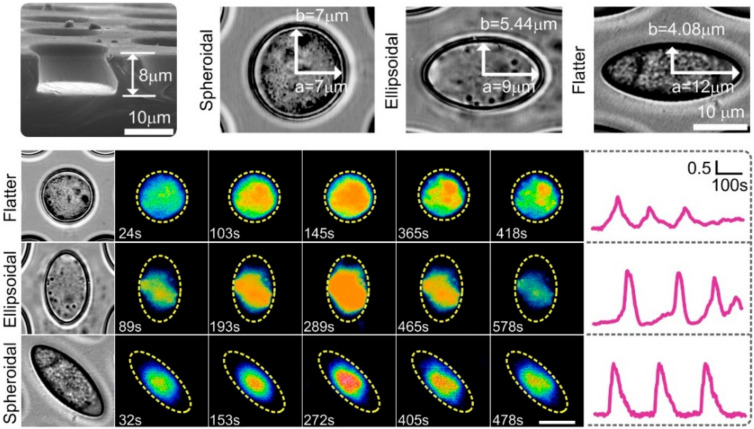
Bright field images of chondrocytes show single cell encapsulated in a microniche with different geometries. Representative single cell images showing Ca^2+^ oscillations at given time points from one chondrocyte in the spheroidala, ellipsoidala, and flattera microniches. Yellow dashed line represents the boundaries of microniche. TRPV4 and PIEZOs may be involved in mediating calcium signaling. Figure based on our recent publication [67].

Topography describes the diverse nanoscale range of the ECM, such as alignment, texture, and nanoscaled patterns on fibrils, as well as the macroscale architecture of the ECM, including shape, organization, and geometry. Our previous results show that the effect of microniche geometry on the frequency and intensity of Ca^2+^ signaling of chondrocytes is completely different from that of the 2D substrate (Figure 4) [67].

Noting that sensing of the mechanical microenvironment by the chondrocytes was dependent on calcium signalling [66]. Thus, microniche geometry is a critical physical cue for regulating calcium signaling. TRPV4 and PIEZOs may participate in mediating calcium signaling during chondrocyte sensing 3D microniche geometry. TRPV4 and PIEZOs may distinctly mediate chondrocyte sensing and respond to the mechanical microenvironment. Recently, a new technique combining pillar arrays and whole-cell patch-clamp was applied to investigate ion channel-mediated mechanoelectrical transduction in primary chondrocytes [43]. This technique follow-up study demonstrated that the modulatory mechanism exhibited by TRPV4 is extremely dependent on the gating stimulus, which is quite distinct from PIEZO1 [68]. Their results showed that the substrate stiffness and disruption of cytoskeletal elements did not modulate TRPV4-mediated currents. However, the activation of PIEZO1 by cell-generated forces is dependent on substrate stiffness and actomyosin traction forces [69,70]. TRPV4 and PIEZO1 with diverse activation and modulatory mechanisms may, to some extent, enable cells to use different pathways to probe their complex and varying mechanical microenvironment and respond accordingly. Thus, these results might reveal a novel understanding of chondrocyte mechanotransduction and therefore be useful for designing cell-instructive scaffolds for functional cartilage tissue engineering.

## 5. Conclusions

Recent investigations represented a major breakthrough for a better understanding of TRPV4 and PIEZO-mediated various mechanotransductions during chondrocyte sensing of the complex microenvironment. The widespread impact of TRPV4 and PIEZOs on various mechanosensory events also raises the possibility that these channels may interact directly or indirectly with other known or unknown proteins to regulate cellular mechanotransduction. Despite the fact that TRPV4 and PIEZOs play an important role in chondrocytes via Ca^2+^ signaling, the identification of other cytokines stimuli-regulated mechanotransduction (inflammatory cytokines, TNF-α, and IL-1β) will undoubtedly shed light on our understanding of pathologic mechanotransduction in OA. Inflammatory signaling sensitizes articular chondrocytes to mechanical trauma.

Understanding the roles of TRPV4 and PIEZOs in the articular cartilage is only the beginning. Thus, it will be necessary to unify disparate fields (mechanobiology, biomaterial, biomolecular engineering, pharmaceutical science, micro- and nanofabrication, and so on) to precisely link TRPV4 and PIEZO-mediated mechanotransduction to the physiology and pathology of articular cartilage. It is undeniable that apart from TRPV4 and PIEZOs, other mechanically activated or mechanosensitive ion channels may also detect mechanical cues and be involved in mechanotransduction processes. Our understanding of which mechanotransduction processes require TRPV4 and PIEZO ion channels and what molecules and processes modulate TRPV4 and PIEZO function is growing rapidly. Yet, there remains much to be done. In particular, little is known about the precise mechanism by which TRPV4 and PIEZOs mediate or are involved in the chondrocyte sensing of the mechanical microenvironment and transduce these mechanical cues into biochemical response. Additionally, there are many mechanotransduction processes for which a role of TRPV4 and PIEZOs has not yet been explicitly tested. Finally, there is little information as to how they mechanistically alter the processes of TRPV4 and PIEZOs in a physiologically relevant microenvironment. One key need going forward is therefore the development of a biomaterial design that can specifically and quantitatively characterize the activity of TRPV4 and PIEZO channels.

In addition, there are major gaps in our understanding of how mechanical microenvironment affects TRPV4 and PIEZO channel-mediated signaling pathways in three dimensions. The question that must be answered is how do TRPV4 and PIEZOs synergistically mediate chondrocyte sensing of the mechanical microenvironment. At present, the exact molecular mechanism of TRPV4 and PIEZO-mediated mechanotransduction during the chondrocyte sensing of mechanical cues remains unknown. Thus, the connection among matrix viscoelasticity and TRPV4 and PIEZO channels, cell signaling, transcription factor activation, and the epigenome is an area ripe for study. Growing findings about TRPV4 and PIEZO-mediated mechanotransduction and mechanosension in chondrocytes will pave the road for developing smart bioresponsive materials capable of recapitulating the physiological microenvironment to initiate a therapeutic response for treating OA. In future study, an engineered microenvironment with a range of specific combinatorial physical and biochemical cues, in concert with defined mechanical stimulation of the high-throughput culture system should be developed to investigate how TRPV4 and PIEZO channels mediate the mechanoregulating and mechanosensing in the chondrocyte microenvironment.

## Data Availability

Not applicable.
